# Expansion of artemisinin partial resistance mutations and lack of histidine rich protein-2 and -3 deletions in *Plasmodium falciparum infections* from Rukara, Rwanda

**DOI:** 10.1186/s12936-024-04981-4

**Published:** 2024-05-16

**Authors:** Cecile Schreidah, David Giesbrecht, Pierre Gashema, Neeva Wernsman Young, Tharcisse Munyaneza, Claude Mambo Muvunyi, Kyaw Thwai, Jean-Baptiste Mazarati, Jeffrey A. Bailey, Jonathan J. Juliano, Corine Karema

**Affiliations:** 1 Brown University, Providence, RI USA; 2 INES-Ruhengeri, Ruhengeri, Rwanda; 3 Rwanda Biomedical Center, Kigali, Rwanda; 4 University of North Carolina at Chapel Hill, Chapel Hill, NC 27599 USA; 5 Quality Equity Health Care, Kigali, Rwanda; 6 Swiss Tropical and Public Health Institute, University of Basel, Basel, Switzerland

**Keywords:** Artemisinin, kelch13, K13, R561H, Rukara, Rwanda, Malaria, *Plasmodium falciparum*, Drug resistance

## Abstract

**Background:**

Emerging artemisinin partial resistance and diagnostic resistance are a threat to malaria control in Africa. *Plasmodium falciparum* kelch13 (*k13*) propeller-domain mutations that confer artemisinin partial resistance have emerged in Africa. *k13*-561H was initially described at a frequency of 7.4% from Masaka in 2014–2015, but not present in nearby Rukara. By 2018, 19.6% of isolates in Masaka and 22% of isolates in Rukara contained the mutation. Longitudinal monitoring is essential to inform control efforts. In Rukara, an assessment was conducted to evaluate recent *k13*-561H prevalence changes, as well as other key mutations. Prevalence of hrp2/3 deletions was also assessed.

**Methods:**

Samples collected in Rukara in 2021 were genotyped for key artemisinin and partner drug resistance mutations using molecular inversion probe assays and for *hrp2/3* deletions using qPCR.

**Results:**

Clinically validated *k13* artemisinin partial resistance mutations continue to increase in prevalence with the overall level of mutant infections reaching 32% in Rwanda. The increase appears to be due to the rapid emergence of *k13*-675V (6.4%, 6/94 infections), previously not observed, rather than continued expansion of 561H (23.5% 20/85). Mutations to partner drugs and other anti-malarials were variable, with high levels of multidrug resistance 1 (*mdr1*) N86 (95.5%) associated with lumefantrine decreased susceptibility and dihydrofolate reductase (*dhfr*) 164L (24.7%) associated with a high level of antifolate resistance, but low levels of amodiaquine resistance polymorphisms with chloroquine resistance transporter (*crt)* 76T: at 6.1% prevalence. No *hrp2* or *hrp3* gene deletions associated with diagnostic resistance were found.

**Conclusions:**

Increasing prevalence of artemisinin partial resistance due to *k13*-561H and the rapid expansion of *k13*-675V is concerning for the longevity of artemisinin effectiveness in the region. False negative RDT results do not appear to be an issue with no *hrp2 or hpr3* deletions detected. Continued molecular surveillance in this region and surrounding areas is needed to follow artemisinin partial resistance and provide early detection of partner drug resistance, which would likely compromise control and increase malaria morbidity and mortality in East Africa.

## Background

Malaria remains a global public health challenge. An estimated 247 million cases and 619,000 deaths occurred worldwide in 2022 with 95% of these cases and 96% of the deaths recorded from the WHO Africa region [1]. The vast majority of deaths occur in children in Africa due to *Plasmodium falciparum*, which accounts for 99% of malaria cases on the continent [2]. The first-line treatment for *P. falciparum* infection is artemisinin-based combination therapy (ACT), which combines a fast-acting artemisinin derivative with a longer-lasting partner drug that effectively eliminates any remaining parasites [3]. But while ACT is a cornerstone for test and treat strategies used throughout Africa, multiple artemisinin partial resistance (ART-R) mutations are now emerging that will likely reduce the effectiveness of treatment, hinder control efforts, potentially further engender partner drug resistance, and lead to eventual ACT clinical failure [4–6].

ART-R manifests as delayed clearance after therapy and is most commonly mediated by various nonsynonymous propeller domain mutations in *pfkelch13* (*k13*) *gene* (*PF3D7_1343700*) [7]. These mutations first emerged over a decade ago and have spread widely in Southeast Asia, along with subsequent partner drug resistance mutations. Together artemisinin and partner drug resistance causes clinical failure and recrudescence after ACT [8]. In the study initially characterizing ART-R in Africa, the Pfkelch13 561H mutation was found in 7.4% of samples from Masaka, Rwanda in 2015. Genomic analysis showed that 561H in Rwanda was genetically distinct in terms of flanking microsatellites haplotypes from previously detected Southeast Asian 561H mutations, providing compelling evidence of a de novo local emergence in Africa [6]. By 2018 in Masaka, the prevalence of 561H had increased dramatically to 19.6% and 561H was also found in 22% of samples from Rukara [9].

**Fig. 1:**
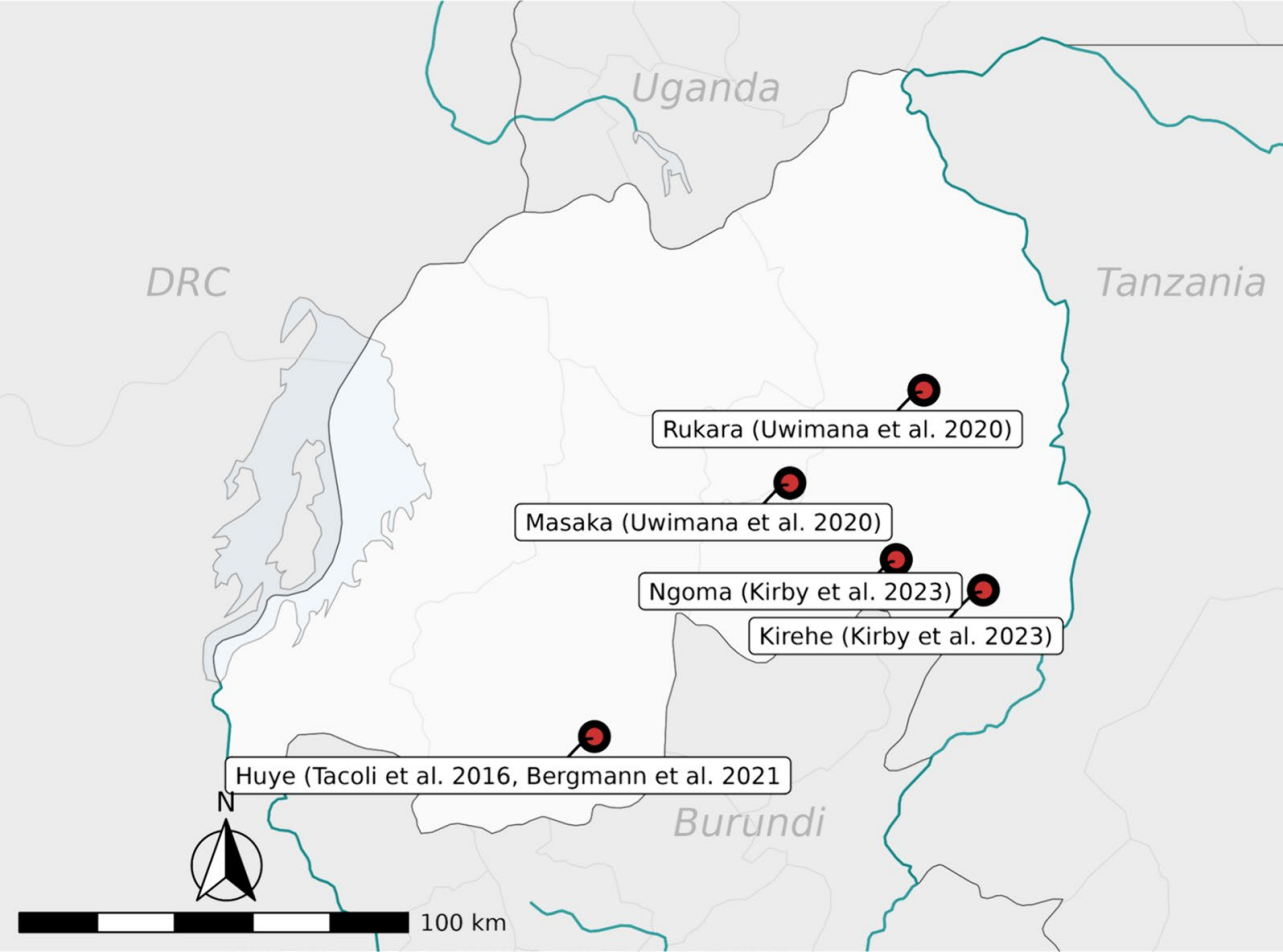
Distribution of Previous reports of K13 Mutations in Rwanda. The map illustrates the geographic distribution of research sites that have reported on K13 mutations, including Kirehe, Ngoma, Huye, Masaka, and Rukara

Further studies have shown that Eastern Africa has become the centre of ART-R emergence with multiple validated *k13* mutations, 469Y (Uganda), 561H (Rwanda), 622I (Eritrea and Ethiopia) and 675V (Uganda), independently emerging and spreading across borders to neighboring countries. In Uganda, clinical studies and detailed prevalence data over time show 675V and 469Y have increased over time and are now highly prevalent in multiple areas [4, 10–14]. In Eritrea and Ethiopia, 622I are increasing in prevalence [5, 15]. The 561H mutation has also appeared now in Uganda and Tanzania [16]. In Rwanda, where 561H was first discovered, current data is more limited. The 561H mutation has been seen at appreciable prevalence in multiple sites (Fig. 1**, **Table 1). In addition, in rare instances other candidate and validated mutations, such as a singular 675V isolate from the Huye district, are reported [9]. However, concerted efforts at repeated or broader sampling are still lacking despite its initial discovery in Rwanda. Additional assessment is urgently needed to better understand emerging ART-R and the impact that 561H mutation may have on anti-malarial therapy.

**Table 1:** Prevalence of Polymorphisms Previously Reported in Rwanda

Mutation	Prevalence, location (year)	Citation
561H	7.4%, Masaka (2015) 5.47%, Kirehe, (2014–2015) 2.47%, Ngoma (2014–2015) 19.6%, Masaka (2018) 22%, Rukara (2018) 4.5%, Huye District (2019)	(6,9) (17) (18)
675V	Singular isolate, Huye District (2015) 4.5%, Huye District (2015)	(13) (18)

Another emerging challenge for test and treat strategies are *P. falciparum* histidine rich protein 2 and 3 (*hrp2/3*) gene deletions. *hrp2* encodes the protein used in most malaria rapid diagnostic tests (RDTs) in Africa, thus deletion of the gene makes the parasite “invisible” or undetectable to RDTs. This diagnostic resistance has emerged and spread in the Horn of Africa and has the potential to emerge elsewhere. Little surveillance data for *hrp2/3* deletions exists in Rwanda. However, given areas of low transmission are of primary concern for the emergence and impact of these mutations, surveillance in Rwanda is likely important [19, 20].

Here, 273 samples were leveraged, collected from uncomplicated malaria infections in Rukara, Rwanda during 2021 routine malaria programme clinical monitoring, to evaluate the status of molecular markers of anti-malarial resistance 3 years from the last measures as well as to assess for the presence of *hrp2/3* gene deletions. Within the dataset, 3 of World Health Organization (WHO) 13 recognized validated *k13* markers of resistance (R561H, P574L, and A675V) were found, but none of their 9 candidate markers [21]. Longitudinal monitoring of *k13* mutations in Rukara helps to provide information about the changing patterns of partial resistance to artemisinin in Rwanda and provides valuable information for modellers and national control programmes interested in studying the spread of polymorphisms that will impact test and treat strategies.

## Methods

### Patient samples

Dried blood spots (DBS) (n = 273) were collected at Rukara Health Centre in 2021 with the intention of evaluating the performance of malaria diagnostics in Rukara, tracking the emergence of *hrp2/3* deletions, and monitoring drug resistance markers. Patients living in the catchment area of Rukara presenting clinical signs and symptoms of uncomplicated malaria with positive RDTs were recruited at the health centre. RDT testing was done using the SD BIOLINE Malaria Ag P.f/Pan test to detect the histidine-rich protein II antigen of *P. falciparum* and pan *Plasmodium* lactate dehydrogenase of *Plasmodium* species in human whole blood. Recruited patients provided whole blood samples by intravenous draw that were used to confirm malaria infection through a malaria smear and the asexual parasite densities were estimated. This study was approved by the National IRB of Rwanda.

### Ethical clearance

The study was approved by the Rwanda National Ethics Committee (IRB00001497). Analysis at Brown University and University of North Carolina was deemed non-human subjects research.

### Molecular inversion probe genotyping

Blood spots were processed using the chelex method [22] to extract DNA and genotyped using molecular inversion probes (MIPs). The DR2 drug resistance MIP panel, as previously detailed [23], was used with no template and no probe negative controls, and positive controls from the reference strains 3d7, Dd2, and HB3 and 7G8. This panel provides data from across *k13*, but also multiple other anti-malarial resistance genes. Mixed isolates were called mutant if alternate read depth was above 4 and UMI redundancy was above 4. Samples were demultiplexed using MIPTools software (available at https://github.com/bailey-lab/MIPTools) and variants were called using the freebayes setting with a minimum of 10 universal molecular identifiers per probe per sample. Further data cleaning and analysis was done in R version 4.1.2 using tidyverse and mapping using sf. Variants were called using the freebayes setting in MIPTools; all packages are detailed here: https://github.com/bailey-lab/Rwanda-DHS-2014-15. Confidence intervals for prevalence were determined using the Binomial exact calculation at https://sample-size.net/confidence-interval-proportion/.

### Molecular detection and determination of *hrp2/3* gene status

All samples were screened by a quantitative real time PCR assay for *P. falciparum* lactate dehydrogenase (*pfldh*) as previously described [24]. A standard curve of mocked dried blood spot samples that used whole blood and cultured parasites (MRA-102, BEI resources, Manassas, VA) was generated. The mocked DBS was extracted in a similar fashion to the clinical samples, allowing us to estimate parasite density of infections after extraction based on real time PCR Ct value relative to the standard curve. For potential *hrp2/3* deletions, samples with a calculated parasitaemia of 100 or more parasites per microlitre were moved forward for *hrp2/3* deletion detection using a multiplexed real time PCR assay as previously described [25]. The use of samples with higher parasitemia is necessary to reduce false positive *hrp2/3* deletion calls [26]. Any sample that did not amplify at any gene, including *hrp2* or *hrp3*, was repeated before a genotype call was made. For qPCR controls, strains 3D7, Dd2, and HB3 were utilized in addition to human DNA Novagen 69237-100UG (Millipore Sigma, Burlington, MA, catalog number 69237–3); negative controls were used in the same manner as previously reported [25].

### Calculations

Using qPCR as the gold standard (Table 3), NPV (negative predictive value), PPV (positive predictive value), sensitivity, and specificity were calculated. NPV was calculated by dividing the amount of true negatives by the sum of true negatives and false negatives and PPV was calculated by dividing the amount of true positives by the sum of true positives and false positives. Then, sensitivity was calculated by dividing the amount of true positives by the sum of true positives and false negatives and specificity was calculated by dividing the amount of true negatives by the sum of true negatives and false positives.

## Results

Of the 273 samples, 135 were successfully genotyped. A summary of all anti-malarial resistance polymorphisms in the samples is shown in Table 2. The 561H mutation was observed at 23.5% (95% CI 15.0–34.0%) prevalence in 2021 compared to the 22% prevalence previously reported in Rukara in 2018 [9]. Furthermore, the 675V mutation, which had previously not been reported on in the Rukara region, was found in 6.4% (95% CI 2.4–13.4%) of isolates. No other validated or candidate ART-R polymorphisms were seen in the samples within the propeller domain, but three other non-synonymous polymorphisms were noted in the area (Table 2) [21]. Furthermore, a common polymorphism K189T, which is outside of the propeller region, was found at 41.7%.

**Table 2:** Prevalence of key drug resistance mutations

Mutation	Prevalence proportion	Confidence interval (95% CI)
K13		
R561H	0.235 (20/85)	0.150–0.340
A675V	0.064 (6/94)	0.024–0.134
P574L	0.024 (2/84)	0.003–0.083
F699C	0.011 (1/87)	0.000–0.062
R575K	0.011(1/88)	0.000–0.062
DHPS		
A437G	0.878 (72/82)	0.787–0.940
K540E	0.784 (69/88)	0.684–0.865
A581G	0.427 (38/89)	0.323–0.536
A613S	0.034 (3/87)	0.007–0.98
S436A	0.000 (0/82)	0.000–0.044*
A613T	0.000 (0/87)	0.000–0.042*
DHFR		
S108N	1.000 (80/80)	0.955–1.000*
N51I	0.934 (85/91)	0.862–0.975
C59R	0.922 (83/90)	0.846–0.968
I164L	0.247 (19/77)	0.156–0.358
MDR1		
N86Y	0.955 (84/88)	0.888–0.988
Y184F	0.717 (66/92)	0.614–0.806
D1246Y	0.979 (95/97)	0.928–0.998
N1042D	0.054 (5/93)	0.18–0.121
CRT		
K76T	0.0610 (5/82)	0.20–0.137

^*^One sided 97.5% CI

**Fig. 2:**
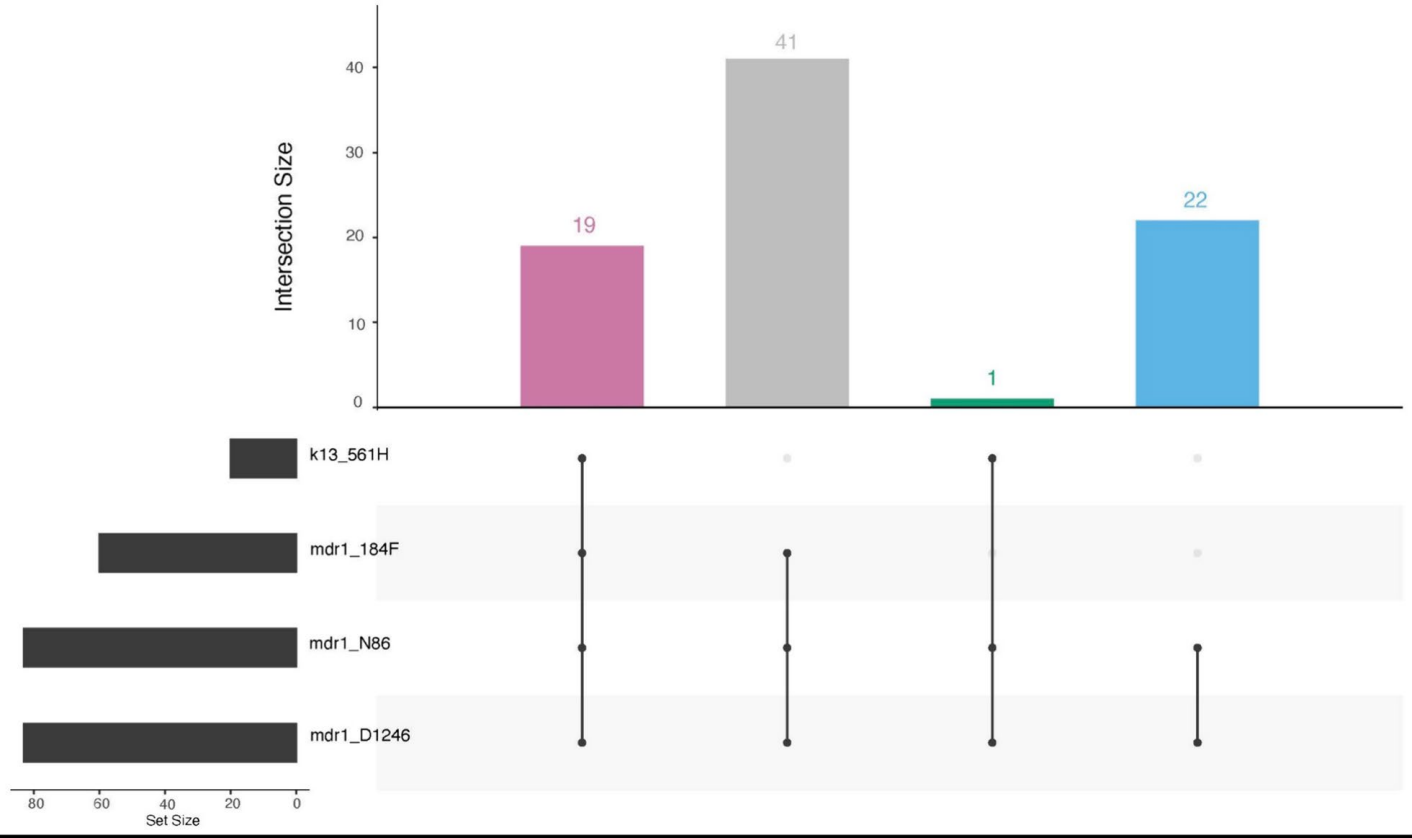
Upset plot of MDR1 and K13 mutations. A total of 82 samples had genotype calls at K13 R561H, MDR1 N86Y, MDR1 Y184F and MDR1 D1246Y. The majority of the parasites had the NFD haplotype in MDR1, with 31.7% (19/60) having the K13 561H mutation

In addition to *k13*, the MIP panel provides data on other anti-malarial resistance polymorphisms. The primary ACT medicine used in Rwanda is artemether-lumefantrine (AL). Having the wild type N86 amino acid of multidrug resistance protein 1 (*mdr1*) has been associated with tolerance to lumefantrine particularly in the context of the NFD (N86, 184F, D1246) haplotype. N86 was near fixation in the population with a prevalence of 95.5% (95% CI 88.8–98.8%). The *mdr1* NFD haplotype was seen in 72.3% (60/83) of isolates where all loci were available. The NFD haplotype did occur in samples with *k13* mutations (Fig. 2). Furthermore, resistance to chloroquine and amodiaquine have been linked to mutations in the chloroquine resistance transporter (*crt*), particularly *crt* 76 T, which was found at a 6.1% prevalence among the samples. This mutation has been associated with better clinical response to artesunate-amodiaquine (ASAQ), which provides Rwanda with a backup drug if AL failure begins to happen [27, 28].

**Fig. 3:**
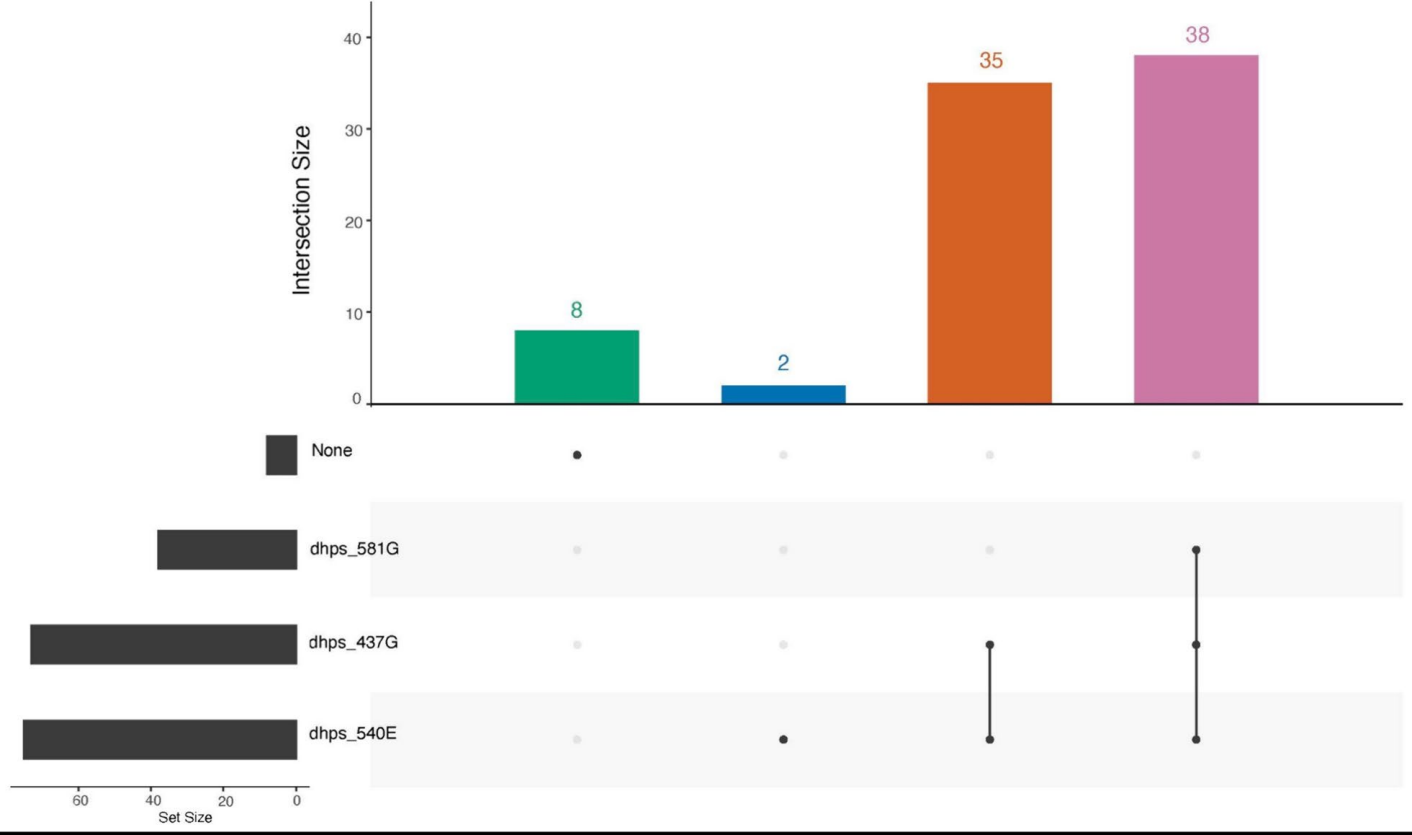
Upset plot of DHPS mutations A total of 83 samples had genotype calls at DHPS A437G, K540E, and A581G and 45.8% (38/83) of the infections had all three mutations, while 88.0% (73/83) had both DHPS 437G and 540E

Resistance to SP occurs through sequential acquisition of mutations in two genes, dihydrofolate reductase (*dhfr*) and dihydropteroate synthase (*dhps*) (Table 2). Importantly, subsequent mutations in haplotypes of *dhfr*, such as 164L and *dhps*, such as 581G, are associated with high-grade pyrimethamine and sulfadoxine resistance, respectively, and significantly reduce the effectiveness of SP used for chemoprevention. Here the *dhfr* 164L mutation can be seen at a prevalence of 24.7% (95% CI 15.6–35.8%) and the *dhps* 581G mutation at a prevalence of 42.7% (95% CI 32.3–53.6%) (Table 2). The *dhps 5*81G mutation occurred in 45.8% (38/83) of samples where data was available for *dhps* 581G, 437G, and 540E mutations (Fig. 3).

**Fig. 4:**
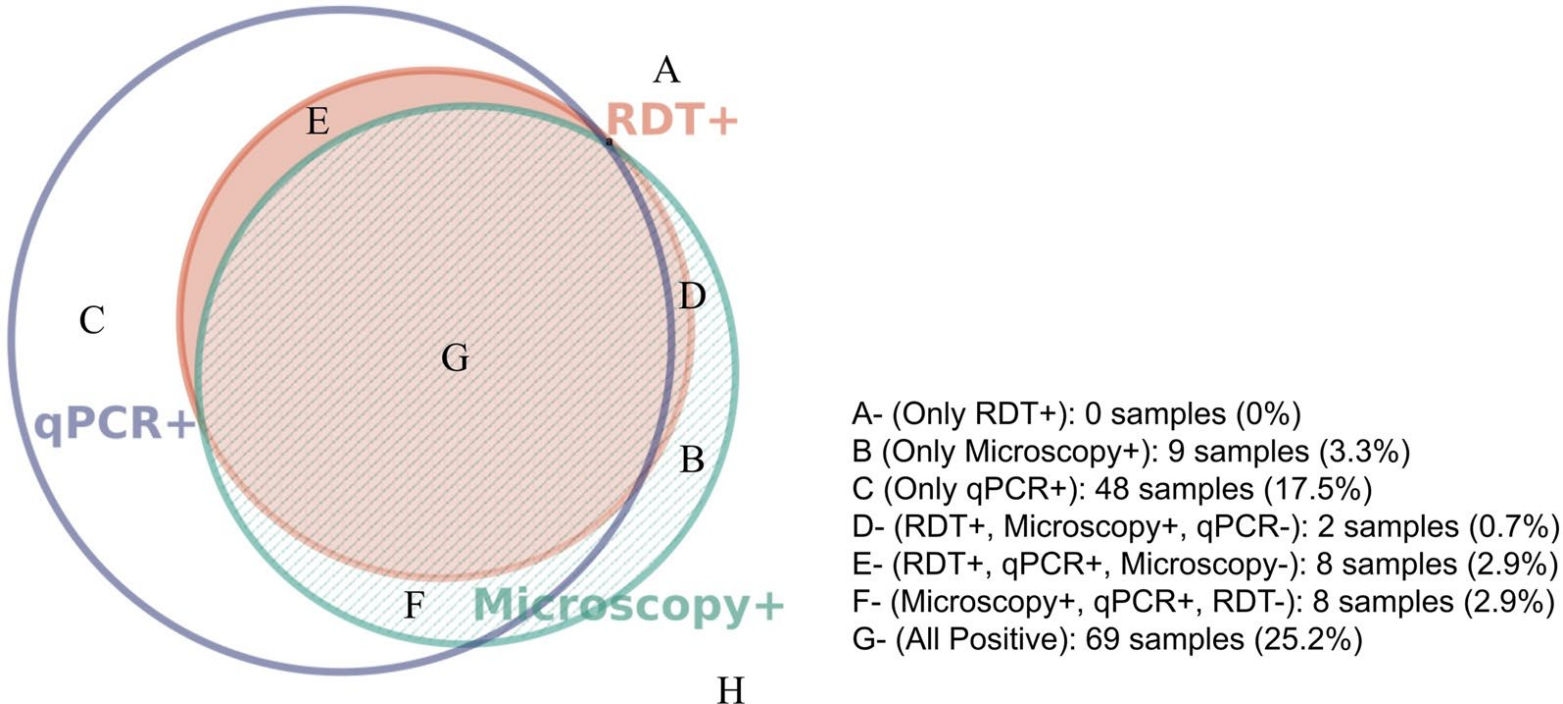
Proportional Euler Venn Diagram Comparing the Three Utilized Diagnostic Methods 130 of the 274 samples (H) were negative for all three tests and are excluded from the Venn diagram [29]

To assss diagnostic performance and evaluate for the presence of *hrp2/3* deletions, 274 samples were examined through RDT, microscopy, and qPCR to assess their positivity; it was observed that 8 samples (Fig. 4) were RDT negative, despite exhibiting positive microscopy and positive qPCR results indicating potential *hrp2/3* deletions in the sample. Of these eight, four had high enough parasitemia to assess for deletions by real time PCR, of which none contained deletions. Using qPCR as the gold standard (Table 3), the RDT had an NPV of 0.71 and a PPV of 0.97, while the blood smear microscopy hadd an NPV of 0.70 and a PPV of 0.88.

**Table 3:** Statistics on RDT and microscopy using qPCR as a gold standard

	RDT	Microscopy
Sensitivity	0.58	0.58
Specificity	0.99	0.92
NPV	0.71	0.70
PPV	0.97	0.88

Sensitivity, specificity, negative predictive value (NPV) and positive predictive value (PPV) are shown as proportions for each diagnostic method

## Discussion

This study provides additional insight into the ongoing emergence of artemisinin partial resistance by providing longitudinal data from one of the sites in Rwanda with early descriptions of *k13* mutations. Importantly, the 675V mutation, previously found in Uganda is reported [4]. Meanwhile, as previously said, 561H appears to be relatively stable in the population (23.5% in 2021 compared to 22% from 2018 [9], and falls within the range modelled for the 561H allele frequency for Rwanda in 2021 [30]. However, the overall level of all validated *k13* mutants has increased to 32% with the arrival of the 675V mutation. The 675V mutation has reached high levels in neighbouring Uganda and the emergence in Rukara likely represents spread across the border given the high-levels of spread within Uganda itself [4, 10]. However, an independent origin can not be ruled out without additional genomic analysis of examining the haplotypes flanking the *k13* gene. In the end, the overall increase in ART-R among parasites is concerning for malaria control in the region.

Beyond *k13*, evidence was found for mutations important for understanding partner drug effectiveness. The combination of high levels of *mdr1* N86 and low *crt* 76 T are reassuring that Rwanda has an effective backup ACT medicine with ASAQ if their first-line should fail. AL continues to have good clinical effectiveness, but high prevalence of the *mdr1* N86 mutation is concerning. The *mdr1* NFD haplotype has been linked to reduced effectiveness towards lumefantrine [31]. The current study found N86 in 95.5% (95% CI 88.8–98.8%), 184F in 71.7% (95% CI 61.4–80.6%) and D1246 in 97.9% (95% CI 92.8–99.8%) of isolates. The NFD haplotype was found in 72.3% (60/83) of samples where all loci were available and this haplotype did occur with *k13* mutations (Fig. 2). This aligns with the results of a recent study in Ethiopia, where the NFD haplotype was found in 83% of the 609 samples collected and co-occurred with the *k13* 622I mutation 98% of the time [5].

SP is routinely used as an intermittent preventive treatment in pregnancy (IPTp) for malaria as well as for seasonal malaria chemoprophylaxis (SMC) and perennial malaria chemoprevention (PMC) in Africa. Historically drug pressure from SP drove the sequential mutations in *dhfr* and *dhps*, but continued pressure from some SP use and activity against malaria parasites by co-trimoxazole (trimethoprim-sulfamethoxazole) may have impacted the prevalence of these mutations [32]. Initially, concerns regarding SP use in the presence of the *dhps* 540E resistance marker arose. Consequently, its usage was only recommended in areas where the marker remained at a low prevalence. However, SP has been shown to be clinically effective even with a high prevalence of *dhps* 540E. Accordingly, WHO guidelines were updated in 2022 to remove restrictions on using SP based on these markers [34]. The SP resistance markers *dhps* 581G and *dhfr* 164L are also associated with high level resistance to [33], raising concern regarding reduced IPTp efficacy. However, current WHO guidelines stress that IPTp-SP should still be used, even with high resistance in a region, as more research is needed to develop more effective alternatives for malaria chemoprevention and determine whether SP restrictions are necessary.

One of these markers, *dhfr* 164L, known to confer heightened resistance to antifolates, had previously been seen with a prevalence of 11% in Rukara between 2001 and 2006 [35]. It was seen at a higher frequency in areas of Uganda, as a 35% prevalence of the mutation was reported in *P. falciparum* isolates in Fort Portal in 2013 [36]. The impact on clinical efficacy of chemoprevention and the evidence of regional spread highlight the need for continued monitoring of these mutations even in countries like Rwanda where IPTp is not used routinely.

Both *dhfr* and *dhps* mutations were common in the study area (Table 2). Notably, *dhps* 540E was identified at a prevalence of 78.4% (95% CI 68.4–86.5%). The *dhps* 581G mutation occurred in 42.7% (95% CI 32.3–53.6%) of isolates, with 45.8% (38/83) of samples, where data from all polymorphisms is available, showing the 437G, 540E, and 581G mutation together (Fig. 3). The presence of late high level antifolate resistance mutations in the region are very concerning, with *dhfr* 164L being found at a prevalence of 24.7% (95% CI 15.6–35.8%). Regional spread of these mutations is a concern and high levels of these mutations have recently been described in North-West Tanzania [16]. An interesting finding is the presence of the *dhps* 613S mutation found at a prevalence of 3.4% (95% CI 0.7–9.8%); this is a mutation typically found in West Africa [37]. However, they have been reported at low frequency as close as neighboring Democratic Republic of the Congo [38]. Overall, this data aligns with previously reported trends in *dhfr* and *dhps* mutations in Rukara where 75% of the isolates tested had three mutations in *dhfr* and two or three in the *dhps* gene [35].

The emergence of *k13* 675V in Rukara is concerning, as it shows the spread of the mutation from Uganda, or potentially an independent origin. While the mutation was reported in the Huye District of southern Rwanda in 2015, no studies have detected it at an appreciable frequency in Rwanda. More concerningly, 675V appears to have increased in frequency more rapidly than 561H suggesting the possible fitness advantage compared to 561H [39]. Future work is needed to understand the relative fitness of the two mutations, but this advantage may be attributable to enhanced survival following treatment with ACT and/or better survival and growth in the absence of drugs. While there are also other *k13* mutations found in the dataset that have not been reported on in Rukara before, namely P574L, F699C, and R575K, their prevalences are all less than 0.03 (3%) and unvalidated which raises questions about their significance and calls for further investigation to determine their impact and potential implications in Rukara.

While this study provides new data on the longitudinal changes in *k13* mutation in Rukara in this study, it is limited in many aspects. These samples represent a convenience sample of participants enrolled in a study not meant to monitor anti-malarial resistance and thus may not be population representative. The geographic scale is also small, and though any longitudinal data for molecular surveillance is valuable, broadly geographically representative sampling reflects the best means for understanding antimalarial resistance trends [4, 10, 40].

*hrp2/3* deletions were not detected in samples discordant by RDT compared to PCR and microscopy. This is reassuring that RDTs that were negative were not due to these deletions, but can be due to other reasons such as lot-to-lot variation, operator error, and poor storage but in this case it is likely due to the fact that *hrp2* is not a perfect correlate with parasitaemia–i.e. every parasitaemia > 100 p/uL may not have enough detectable *hrp2* [20, 41]. A previous report from 2017 by Kozycki et al*.* [42] found low levels of PCR confirmed *hrp2* deletion (32/3291). These results suggest that *hrp2/3* deleted parasites are not increasing in prevalence since that time, though the sample size is too small to conclusively confirm a change in prevalence.

## Conclusion

The results of this study support that artemisinin partial resistance, partner drug resistance, and resistance to anti-malarials used for antifolate chemoprophylaxis are all emerging and will pose imminent challenges for malaria control programmes in Africa. Longitudinal molecular surveillance is needed for addressing these concerns in a timely manner and to help inform policy. Effective molecular surveillance can help rapidly inform where either in vitro*/*ex vivo assessment resistance or therapeutic efficacy studies can be targeted. Emergence of *hrp2/3* deletions in Africa may also eventually pose a challenge outside of the Horn of Africa. Fortunately *hrp2/3* deletions do not appear to be a problem in Rwanda at this time. Together, this information provides valuable data for the national malaria control programme and for modelers and other scientists interested in studying the emergence and spread of anti-malarial resistance.

## Data Availability

Data is available upon reasonable request to the corresponding author.
